# Clinical Score for Predicting the Risk of Poor Ambulation at Discharge in Fragility Femoral Neck Fracture Patients: A Development Study

**DOI:** 10.3390/jcm11164871

**Published:** 2022-08-19

**Authors:** Paween Tangchitphisut, Jiraporn Khorana, Jayanton Patumanond, Sattaya Rojanasthien, Theerachai Apivatthakakul, Phichayut Phinyo

**Affiliations:** 1 Department of Orthopaedics, School of Medicine, Mae Fah Luang University, Chiang Rai 57100, Thailand; 2 Department of Surgery, Faculty of Medicine, Chiang Mai University, Chiang Mai 50200, Thailand; 3 Center for Clinical Epidemiology and Clinical Statistics, Faculty of Medicine, Chiang Mai University, Chiang Mai 50200, Thailand; 4 Department of Orthopaedics, Faculty of Medicine, Chiang Mai University, Chiang Mai 50200, Thailand; 5 Department of Family Medicine, Faculty of Medicine, Chiang Mai University, Chiang Mai 50200, Thailand; 6 Musculoskeletal Science and Translational Research (MSTR) Cluster, Chiang Mai University, Chiang Mai 50200, Thailand

**Keywords:** femoral neck fractures, risk factors, risk scores, probability, rehabilitation

## Abstract

Surgical treatment in patients with fragility femoral neck fractures often leads to a longer length of hospital stay (LOS) and higher costs. Intensive rehabilitation is one of the choices to reduce LOS, but patient selection criteria are controversial. We intended to develop a clinical score to predict the risk of poor ambulation at discharge. This study was based on a retrospective cohort of patients diagnosed with fragility femoral neck fractures surgically managed from January 2010 to December 2019 at Chiang Mai University (CMU) Hospital. Pre-, intra-, and post-operative factors that affect rehabilitation training were candidate predictors. All patients were categorized into able or unable groups based on their ability to bear self-weight at discharge. Logistic regression was used for score derivation. Five hundred and nine patients were included in this study. Male sex, end-stage kidney disease (ESRD), cerebrovascular disease, psychiatric disorders, pre-fracture ambulation with gait aids, concomitant fracture, post-operative intensive care unit (ICU) admission or ventilator use, and urinary catheter use at second day post-operation were identified as the prognostic factors. The score showed an AuROC of 0.84 with good calibration. The score can be used for risk stratification on the second day post-operation. External validation is encouraged before clinical implementation.

## 1. Introduction

Currently, surgical management is the primary treatment for clinically stable fragility hip fractures, generally defined as any fracture in an adult over 50 that occurs by a low-energy mechanism of injury [1,2]. However, surgical management often leads to a longer length of hospital stay (LOS) [3,4,5], which is significantly associated with higher morbidity, mortality, and healthcare costs [6,7]. Several strategies have been studied to decrease the LOS. However, clear recommendations have not been settled on. Early surgery and rehabilitation have been proposed to reduce the overall LOS among geriatric hip fracture patients [2,4,8,9,10]. However, certain barriers exist in practice and hinder the effective implementation of early in-hospital rehabilitation, such as delayed surgery, insufficient mobilization, functionally independent training, a lack of coordinated discharge planning or referral, and family or caregiver burdens [11].

Stratifying patients according to their risk of poor ambulation at discharge may lead to better prioritization and reduce the aforementioned barriers. The intensive rehabilitation program should only focus on patients who are unlikely to achieve adequate ambulation within their hospital stays. Our previous study in patients with fragility hip fractures reported prognostic factors that were predictive of the inability to self-ambulate at discharge, such as patients with significant comorbidities, impaired baseline ambulatory status, associated fractures, and pressure ulcers [12]. Recognizing these factors during patient evaluation could potentially help clinicians to identify patients at risk of poor ambulatory status at discharge; however, this approach does not consider the multivariable nature of clinical prediction and does not provide estimations of the individual probability of poor ambulation.

A multivariable prediction score is an attractive clinical tool that simultaneously considers multiple features within each patient and provides absolute predictive values of outcomes. To date, only a few clinical scores for predicting in-hospital ambulation status among geriatric hip fracture patients are available, and often they are not specific for each subtype of hip fracture, resulting in low predictive performance [13]. Moreover, some of the previous scores did not include factors that were associated with the functional outcomes of the patients, such as concomitant fracture, post-operative complications, and post-operative intensive care unit (ICU) admissions [14,15,16,17,18,19], but incorporated certain factors that were not routinely collected in practice, such as the mental status examination (MSE), and, thus, were irrelevant and impractical [20]. In this study, we intended to develop a simplified clinical score to predict the risk of poor ambulation or being unable to bear self-weight at discharge in patients with fragility femoral neck fractures. The information gained from the score might be useful for clinicians to predict early post-operative functional outcomes and provide optimal, individualized rehabilitation plans for each patient.

## 2. Materials and Methods

### 2.1. Study Design

A clinical risk score was developed based on a retrospective observational cohort of patients diagnosed with fragility femoral neck fractures surgically managed with fixation or arthroplasty from January 2010 to December 2019 at Chiang Mai University (CMU) Hospital, a tertiary care center, and medical school. The study protocol was approved by the Research Ethics Committee, Faculty of Medicine, Chiang Mai University (certificate of approval No. EXEMPTION 7375/2020). Patient consent was waived due to the retrospective nature of data collection.

### 2.2. Study Patients

Adult patients with fragility femoral neck fractures who were operated on at our institution during the study period were included. Fragility neck fracture was defined as any fracture in an adult over 50 that occurred by a low-energy mechanism of injury (e.g., falling from a standing height). The exclusion criteria were patients who were referred to or from other hospitals and patients with poor baseline ambulatory status (ambulation with wheelchairs or non-ambulatory status, e.g., bedridden).

### 2.3. Candiate Predictors and Data Collection

We collected data on evidence-proven pre-, intra-, and post-operative factors that affect rehabilitation training and ambulation status at discharge. These factors were used as candidate predictors during statistical modeling and score derivation. The pre-operative factors were gender [14,15], age [14,21], body mass index (BMI) [22], pre-injury ambulation status [13,21,23,24], serum albumin level [25,26], concomitant fracture [27], second hip fracture [28], operative technique (arthroplasty or fixation surgery) [29], and any comorbidity that might affect patients’ rehabilitation and ambulation [30,31]. The intra-operative factors were the amount of time from admission to surgery, total anesthesia time, and volume of intra-operative blood loss [32]. Lastly, the post-operative factors included post-operative intensive care unit (ICU) admission or ventilator use, post-operative sedative drug use, pain score on the initial rehabilitation day [33], post-operative blood transfusion [34], and urinary catheter use on the second day post-operation [35].

### 2.4. Study Endpoint

The endpoint to be predicted was the ambulatory status at discharge for each patient. All patients were categorized based on their ability to bear self-weight at discharge as either able or unable. Patients who could not bear self-weight were defined as those who could only ambulate in a wheelchair or those who could not ambulate or were bedridden. In the other group, patients who were able to bear self-weight were defined as patients who had independent ambulation or patients who could ambulate with gait aids (crutch, cane, or walker).

### 2.5. Sample Size Estimation

We estimated the sample size required for developing the multivariable clinical risk score using methods suggested by Riley et al. [36]. Assuming that the number of significant candidate predictors from the univariable analysis was 15 and estimating the concordance statistics to be 0.85 and the average incidence of the outcome to be 20% based on our previous report [12], a minimum sample size of 460 with 92 events was needed. Achieving this target sample size would guarantee a 0.05 acceptable difference in apparent and adjusted R-squared, a 0.05 margin of error in the estimation of intercept, and an optimal number of events per predictor.

### 2.6. Statistical Analysis

All data analyses were performed using Stata 17 (StataCorp, College Station, TX, USA). The level of statistical significance was considered to be a *p*-value less than 0.05. Frequency and percentage were used to describe categorical variables. Mean and standard deviation (SD) or median and interquartile range (IQR) were used to describe numerical variables based on the underlying distribution. A comparison of categorical variables between groups was conducted with Fisher’s exact test. Student’s t-test or the Mann–Whitney U test was used to compare numerical variables. The area under the receiver operating characteristic (AuROC) curve for each candidate predictor was also presented to reflect the discriminative performance [37].

#### 2.6.1. Score Development

Association between candidate predictors and outcome was explored using univariable binary logistic regression. Missing data were explored. If any predictor showed more than 50% missing data, that predictor was not included in the multivariable analysis. Multiple imputation (MI) with the chained equation method (MICE) was used to handle the missing data and improve statistical efficiency. Gender, age, pre-fracture ambulation status, underlying diseases, operative technique, and ambulation status at discharge were pre-selected as independent variables to predict the missing values using linear regression.

Multivariable binary logistic regression with a stepwise backward elimination method was used to identify the final set of predictors. Predictors with a *p*-value less than 0.05 in the multivariable model were removed in a stepwise manner. After the final model was derived, the weighted score of each remaining predictor was calculated by dividing each logit coefficient by the lowest one and rounded up to the nearest integer.

#### 2.6.2. Model Performance and Internal Validation

The model performance was assessed in terms of discrimination and calibration. The discriminative ability was represented with an AuROC. The score calibration was illustrated with the score calibration plot. Internal validation of this model was carried out via bootstrap re-sampling with 1000 replicates. The test AuROC, calibration slope, and the bootstrap shrinkage factor were reported.

#### 2.6.3. Categorizing Risk Groups

For clinical applicability, the newly derived risk score was categorized into three groups: low, moderate, and high risk of inability to bear self-weight at discharge. The cut-point was determined by considering both clinical and statistical aspects of the clinical decision in this context. The lower and the higher cut-off points were chosen based on the sensitivity, specificity, and likelihood ratio (LR) of each possible score cut-off point. The low-risk group had high sensitivity and the upper bound of the 95% CI of an LR less than 1, whereas the high-risk group had high specificity and the lower bound of the 95% CI of an LR higher than 5. The moderate risk group had an LR ranging from 1 to 10 [38].

## 3. Results

### 3.1. Study Patients

**Figure 1:**
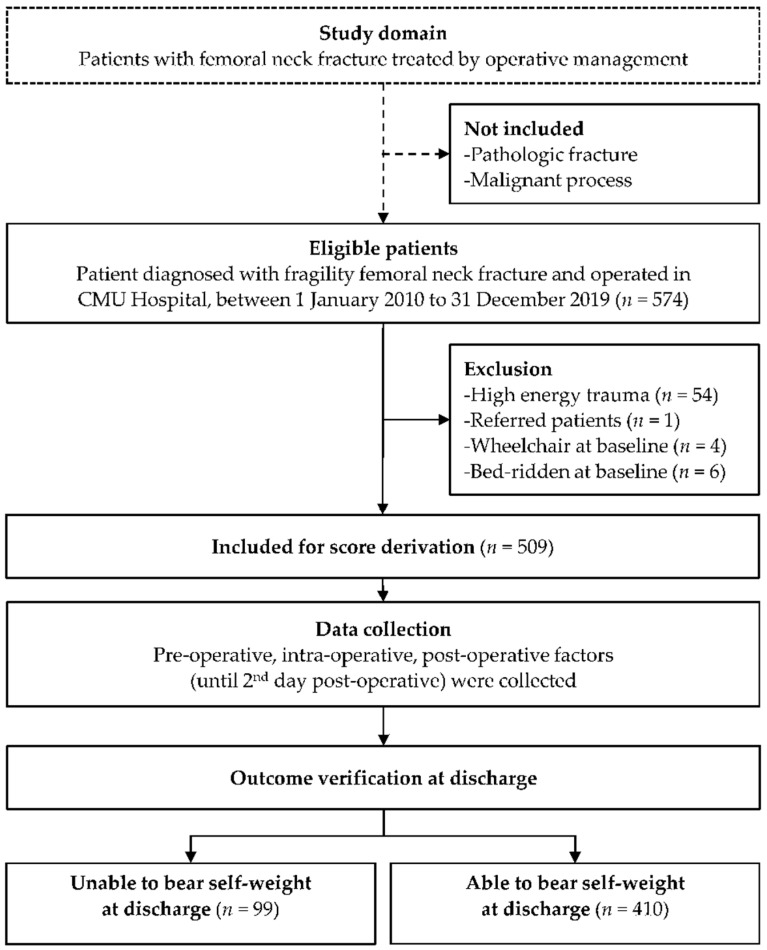
Study flow diagram of the patient cohort.

Five hundred and seventy-four patients with fragility femoral neck fractures were eligible for inclusion. Sixty-five patients were excluded: fifty-four were injured due to high-energy trauma, six were bedridden at baseline, four used wheelchairs at baseline, and one patient was referred to another hospital. Finally, 509 patients were included for statistical analysis and score derivation (Figure 1). Of these, 99 (19.4%) patients were unable to bear self-weight at discharge. In this cohort, most of the patients (408, 80.2%) underwent surgical arthroplasty (396 (77.8%) hemiarthroplasty and 12 (2.4%) total hip arthroplasty), while only 101 (19.8%) received surgical fixation (73 (14.3%) multiple screw fixation and 28 (5.5%) dynamic hip screw fixation).

### 3.2. Candidate Predictors

Table 1 describes and compares the differences in baseline characteristics and pre-, intra-, and post-operative factors between patients who were unable and able to bear self-weight at discharge. There were significant differences between the two groups in several predictors (Table 1). The AuROC analysis identified four prognostic factors that had an AuROC ≥ 0.60, which were gender, pre-fracture ambulation status, hypoalbuminemia, and urinary catheter use on the second day post-operation. Table 2 shows the results of univariable and multivariable logistic regression. A total of twelve candidate predictors with statistical significance according to the univariable analysis were included in the multivariable analysis (Table 2).

### 3.3. Multivariable Modeling and Score Derivation

After stepwise backward elimination, eight predictors remained significant. They were used for score derivation: male gender, ESRD, cerebrovascular disease, psychiatric disorders, pre-fracture ambulation with gait aids, concomitant fracture, post-operative ICU admission or ventilator use, and urinary catheter use on the second day post-operation (Table 3). The logit coefficient of each predictor was transformed into the weighted score by dividing each logit coefficient by the lowest one (0.96) and rounded up to the nearest integer, as shown in Table 3. The possible total score ranged from 0 to 14.5 points. For our data, the scores ranged from a minimum of 0 to a maximum of 8.5. The higher the score, the higher the probability of inability to bear self-weight at discharge. The score was significantly different between the two groups (median 3 (IQR 2.5, 5) vs. median 1 (IQR 0, 2), *p* < 0.001).

### 3.4. Model Performance and Internal Validation

**Figure 2:**
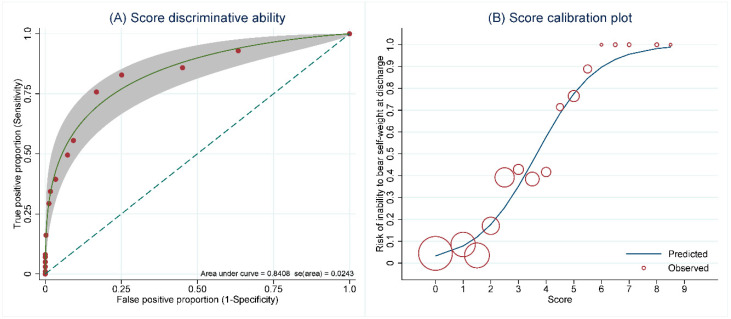
Apparent validation of score performance in terms of discrimination and calibration. (**A**) parametric receiver operating characteristic (ROC) curve representing the ability of the score to discriminate between patients who were able and unable to bear self-weight at discharge, (**B**) calibration plot visualizing the agreement between the predicted risk and the observed proportion of the outcome across the range of the newly-derived score.

Our clinical risk score showed excellent discriminative performance with an apparent AuROC of 0.84 (95% CI 0.79, 0.89) (Figure 2A). The score also showed good agreement between the predicted probability of the inability to bear self-weight at discharge and the observed proportion of patients with the outcomes according to the score calibration plot (Figure 2B).

Bootstrap internal validation reported a test AuROC of 0.83 (95% CI 0.78, 0.88). The calibration slope was 0.92 (95% CI 0.75, 1.14), and the bootstrap shrinkage factor was 0.923.

### 3.5. Risk Categories and Score Accuracy

Two cut-off points were identified, a lower cut-off point at a score > 1 and a higher cut-off point at a score ≥ 4. At the lower cut-off point, the sensitivity was 92.9%, and the specificity was 36.6%, whereas the sensitivity was 39.4%, and the specificity was 96.6% for the higher cut-off point. Patients with risk scores of 0 to 1 were classified as low-risk, those with risk scores of 1.5 to 3.5 were classified as moderate-risk, and those with a risk score higher or equal to 4 were classified as high-risk. The positive predictive values and the LR of each risk category are shown in Table 4.

## 4. Discussion

This study developed and internally validated a simplified and fracture-specific clinical risk score for predicting the inability to bear self-weight at discharge in patients with fragility femoral neck fractures. The newly developed score consists of eight clinically relevant and routinely available predictors: gender, ESRD, cerebrovascular disease, psychiatric disorders, pre-fracture ambulation with gait aids, concomitant fracture, post-operative ICU admission, or ventilator use, and urinary catheter use on the second day post-operation. The score showed excellent discriminative ability and good calibration. Based on the internal validation, the degree of optimism, or overfitting, was low.

Currently, clinicians often rely on standard physical performance scores or individualized multivariable risk scoring systems to predict post-operative ambulation status in patients with fragility hip fractures [18]. The commonly used standard physical performance scores in practice were Barthel-20 [39], Barthel-100 [40], Cumulated Ambulatory Score (CAS) [19], and New Mobility Score (NMS) [15]. However, these scores were not specifically designed to predict ambulatory status in geriatric patients with hip fractures, which explains why their discriminative performances in validation studies were often low, with an AuROC ranging from 0.64 to 0.66 [13]. In contrast, individualized risk scoring systems have continuously emerged as a more attractive and more specific method that can improve predictive performance [17,18,19]. A few scoring systems have been developed for the prediction of post-operative functional outcomes and functional statuses, such as the simple scoring system by Hagino T. et al. [17], the six risk scores by Burgos E. et al. [23], the prognostic model for predicting the recovery of walking independence of elderly patients after hip-fracture surgery by Bellelli G. et al. [41], and the predictive model of gait recovery at one month after hip fracture [16]. Table 5 provides specific details of the study domain, predictors used, and the definition of the clinical endpoint for each scoring system.

The American Society of Anesthesiologists’ (ASA) Physical Status Classification, the Charlton Comorbidity Index (CCI), and the Mental State Examination (MSE) were commonly incorporated within these scoring methods, as mentioned earlier because they represent the comorbidity burden of the patients. However, these parameters have some important weaknesses. Firstly, the ASA classification shows fair interrater reliability, which may result in an inconsistent assessment [42]. Secondly, several comorbidities that might significantly affect the rehabilitation outcomes, such as psychological disorders, are not included in the CCI [43]. Thirdly, the MSE is a standard tool for evaluating the neurocognitive status of geriatric patients [44] and may be able to identify patients who would not respond well to rehabilitation. However, due to its low sensitivity, the MSE might not be appropriate for screening [20]. Moreover, most hospitals in Thailand do not perform MSE screening in their routine outpatient and inpatient practice. Finally, these scoring systems do not include some well-known predictive factors that serve as significant barriers to post-operative rehabilitation (e.g., concomitant fracture, post-operative ICU admission or ventilator use, and post-operative complication) [14,15,16,17,18,19]. Surgical techniques might be another promising prognostic factor that could have good predictive ability and should be incorporated into clinical scoring systems. Hip arthroplasty with the cementing technique has been proven to result in less post-operative pain and early postoperative ambulation due to immediate implant stability [29]. In contrast, patients who were treated by surgical fixation have to ambulate by protective weight bearing during the early phase because early full weight bearing may lead to several complications, such as excessive hip screw sliding, hip screw breakdown, and the hip screw cutting out from the femoral head [45].

In this study, we developed a simplified and practical clinical risk scoring system for patients with fragility femoral neck fractures using readily available clinical data. The specificity of the domain and the inclusion of clinically relevant predictors and predictors that affect post-operative rehabilitation outcomes led to the high predictive ability of the score. The point of prediction of the score was defined on the second day post-operation, which is generally an appropriate time for patient evaluation, as common post-operative events, including the removal of urinary catheters, post-operative complications, and ICU admissions, usually occur within 48 h after surgery [46]. For clinical applicability, the score was categorized into three risk groups: low-risk (0–1 points), moderate-risk (1.5–3.5 points), and high-risk (≥4 points).

**Figure 3:**
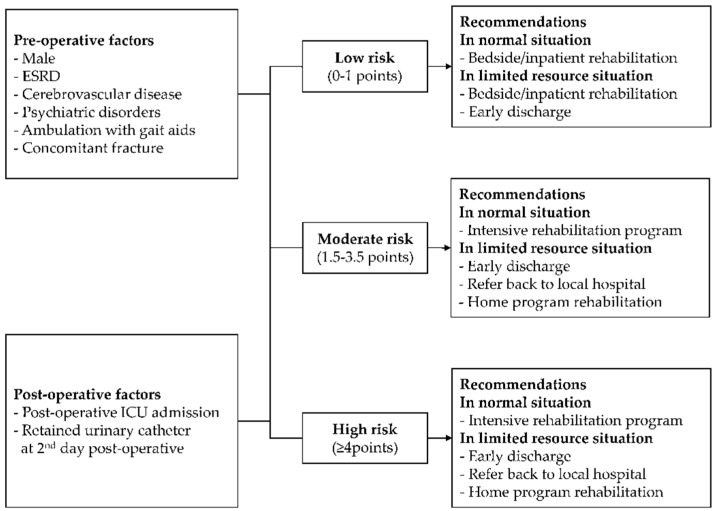
Recommendations for clinical and rehabilitation management according to each patient risk group in regular and limited resource situations.

Intensive rehabilitation aims to minimize impairments and improve functional independence as much as possible [47]. However, the definitive criteria of patients who are candidates for intensive rehabilitation are controversial and remain unclear. In Thailand, the indication for intensive rehabilitation is left either to the discretion of the attending physicians or the local policy and practice guidelines of each hospital [4,9,10]. This ambiguity leads to improper rehabilitation consultation and management, prolonged LOS, and higher overall healthcare costs. Our newly developed clinical risk score confers potential clinical benefits in helping clinicians to properly stratify their patients into three separated risk groups according to their risk of inability to bear self-weight at discharge. We recommend that intensive rehabilitation programs be offered only to patients in the high-risk group, whereas early or usual in-ward rehabilitation can be offered to both the low- and moderate-risk groups (Figure 3). Unpredictable external factors could, however, affect the hospital’s capacity for the effective delivery of the rehabilitation program. The COVID-19 pandemic is a good example of a critical situation that could tremendously limit the capacity of hospital services [48]. In a limited resource situation, patients with hip fractures should be discharged early from the hospital [49], as these patients are often accompanied by high-clinical-risk features for COVID-19 pneumonia and mortality, such as older age and multiple comorbidities [50,51]. Thus, patients predicted to be moderate- to high-risk should be candidates for early discharge and home-based rehabilitation (Figure 3). In addition, effective planning and the training of caregivers for home-based rehabilitation showed no difference in short-term outcomes compared to intensive in-patient rehabilitation [52]. Undoubtedly, the implementation of our score should be further modified to fit the organizational context of each hospital.

Our study has important strengths and limitations. In terms of strengths, firstly, the development data included a homogenous population domain that directly answers our clinical question. Secondly, all predictors incorporated within the model were evidence-based, clinically relevant, and routinely available. Thirdly, the sample size and the number of events in this cohort were adequate for multivariable score development. The most important limitation of our study was the data collection design, which was retrospective. Thus, the quality of data might not be perfect, and missing data for some important predictors may have affected the model’s statistical power and caused biases. In this study, missing data were properly handled using standard multiple imputation methods to maximize the amount of information used during statistical modeling and preserve statistical power. Secondly, the point of prediction on the second day post-operation may be too delayed to guide clinical and rehabilitation management in some circumstances. Ideally, predictions using only pre- and intra-operative factors might be more feasible in actual practice. Thirdly, the number of predictors included was high and may affect clinical applications. Additional efforts might be taken to further simplify the score by reducing the number of predictors or improve the practicality by embedding the score within a web or mobile application. Fourthly, our patient cohort only included Thai patients who visited a single tertiary care center and may only represent Thailand’s specific clinical context. An external validation study is therefore required before clinical implementation in different settings. Finally, the predicted outcome of our scoring system was dichotomized as either able or unable to ambulate at discharge, as we believed it would be sufficient for patient and family counselling in the acute phase. During this phase, the patients and their family members, or caregivers, are often anxious about the prognosis after treatment and require effective risk communication and short- and long-term treatment plans from clinicians [53]. Moreover, the post-operative status could be altered during home-program ambulation training. It was revealed that about 1/3 of the patients could return to pre-fracture mobility or functional independence, and about 1/2 to 2/3 of the patients could achieve activities of daily living (ADL) without difficulty [54]. Further research could develop a prediction model to estimate the probability of each ambulatory level, such as partial weight bearing, full weight bearing, non-weight bearing, or unable to ambulate, which might provide more useful information for shared decision making. However, a larger sample size and a well-balanced proportion of each ambulatory status are needed to yield optimal estimates.

## 5. Conclusions

A simplified clinical risk score was developed to predict the probability of inability to bear self-weight at discharge in patients with fragility femoral neck fractures. The score incorporated eight clinical predictors and can be used for prediction and risk stratification on the second day post-operation. Recommendations were provided for group-specific rehabilitation management protocols during admission, with the primary intention of optimizing patient functional outcomes and reducing the overall LOS and healthcare cost. Although the score showed excellent discriminative ability and good calibration within the development dataset, validating the score in other external datasets is highly encouraged before clinical implementation.

## Figures and Tables

**Table 1:** Baseline characteristics and pre-, intra-, and post-operative factors of patients with fragility femoral neck fractures included in this cohort by their ability to bear self-weight at discharge.

	Missing Value *n* (%)	Unable to Bear Self-Weight at Discharge (*n* = 99) *n* (%)	Able to Bear Self-Weight at Discharge (*n* = 410) *n* (%)	*p*-Value	AuROC (95% CI)
Gender					0.60
- Male - Female	0 (0)	44 (44.44) 55 (55.56)	100 (24.39) 310 (75.61)	<0.001	(0.55, 0.65)
Age ≥ 80 years	0 (0)	52 (52.53)	183 (44.63)	0.178	0.54 (0.48, 0.59)
BMI ≥ 25 kg/m^2^	86 (16.90)	14 (14.14)	61 (14.39)	1.000	0.50 (0.45, 0.55)
Comorbidity					
- ESRD	0 (0)	27 (27.27)	57 (13.90)	0.002	0.57 (0.52, 0.61)
- Cirrhosis	0 (0)	4 (4.04)	0 (0)	0.001	0.52 (0.50, 0.54)
- Cerebrovascular diseases	0 (0)	19 (19.19)	33 (8.05)	0.003	0.56 (0.51, 0.60)
- Psychiatric disorders ^a^	0 (0)	10 (10.10)	15 (3.66)	0.016	0.53 (0.50, 0.56)
- Parkinson’s disease	0 (0)	4 (4.04)	11 (2.68)	0.506	0.51 (0.49, 0.53)
- Diabetes mellitus	0 (0)	21 (21.21)	88 (21.46)	1.000	0.50 (0.45, 0.54)
- Heart diseases	0 (0)	23 (23.23)	66 (16.10)	0.105	0.54 (0.49, 0.58)
- COPD ^b^/asthma	0 (0)	10 (10.10)	28 (6.83)	0.287	0.51 (0.48, 0.55)
- Eye diseases ^c^	0 (0)	10 (10.10)	42 (10.24)	1.000	0.50 (0.47, 0.53)
- Cancer	0 (0)	5 (5.05)	29 (7.07)	0.654	0.49 (0.46, 0.51)
- Dementia	0 (0)	8 (8.08)	31 (7.56)	0.835	0.50 (0.47, 0.53)
Pre-fracture ambulation status					
- Independent ambulation - Ambulation with gait aids	0 (0)	51 (51.52) 48 (48.48)	306 (74.63) 104 (25.37)	<0.001	0.62 (0.56, 0.67)
Hypoalbuminemia (<3.5 g/dL)	92 (18.07)	60 (60.61)	170 (41.46)	0.001	0.56 (0.53, 0.60)
Concomitant fracture	0 (0)	15 (15.15)	4 (0.98)	<0.001	0.57 (0.54, 0.61)
Second hip fracture	0 (0)	11 (11.11)	26 (6.34)	0.128	0.52 (0.49, 0.56)
Operative technique	0 (0)			0.160	0.53 (0.49, 0.58)
- Arthroplasty ^d^ - Fixation ^e^		74 (74.75) 25 (25.25)	334 (81.46) 76 (18.54)		
Intra-operative factors					
- Time delayed from admission to surgery >48 h	0 (0)	95 (95.96)	351 (85.61)	0.003	0.55 (0.53, 0.58)
- Anesthesia time (hours) *	0 (0)	2 (1.75, 2.42)	1.92 (1.67, 2.25)	0.010 ‡	0.58 (0.52, 0.64)
- Intra-operative blood loss (mL) *	0 (0)	100 (50, 200)	100 (100, 200)	0.568 ‡	0.48 (0.42, 0.55)
Post-operative factors					
- Post-operative ICU admission or ventilator use	0 (0)	19 (19.19)	9 (2.20)	<0.001	0.59 (0.55, 0.62)
- Post-operative sedative drug use	0 (0)	41 (41.41)	113 (27.56)	0.010	0.57 (0.52, 0.62)
- Post-operative blood transfusion	0 (0)	31 (31.31)	106 (25.85)	0.312	0.53 (0.48, 0.58)
- Urinary catheter use on the second day post-operation	0 (0)	50 (50.51)	90 (21.95)	<0.001	0.64 (0.59, 0.70)
- Moderate to severe pain score on the rehabilitation day (PS = 4–10)	27 (5.30)	12 (13.48)	78 (19.85)	0.178	0.47 (0.43, 0.51)

‡ Mann–Whitney U test; * median (IQR); ^a^ included patients with drug abuse; ^b^ COPD = chronic obstructive pulmonary disease; ^c^ eye diseases included blindness, cataracts, and glaucoma; ^d^ arthroplasty included total hip and hemiarthroplasty; ^e^ fixation included multiple screw fixation and dynamic hip screw fixation (DHS).

**Table 2:** Univariable and multivariable association between pre-, intra-, and post-operative predictors and the inability to bear self-weight at discharge in patients with fragility femoral neck fractures.

	uOR	95%CI	*p*-Value	mOR	95%CI	*p*-Value
Male	2.48	1.57, 3.91	<0.001	3.18	1.79, 5.66	<0.001
Age ≥ 80 years	1.37	0.88, 2.13	0.158		(Not included)	
BMI ≥ 25 kg/m^2^	0.99	0.52, 1.84	0.949		(Not included)	
Comorbidity						
- ESRD	2.32	1.38, 3.92	0.002	6.37	2.67, 15.20	<0.001
- Cerebrovascular diseases	2.71	1.47, 5.01	0.001	3.68	1.73, 7.88	0.001
- Psychiatric disorders ^a^	2.96	1.29, 6.80	0.011	4.10	1.43, 11.75	0.009
- Parkinson’s disease	1.53	0.48, 4.90	0.477		(Not included)	
- Diabetes mellitus	0.99	0.58, 1.68	0.956		(Not included)	
- Heart diseases	1.58	0.92, 2.70	0.095		(Not included)	
- COPD ^b^/asthma	1.53	0.72, 3.27	0.269		(Not included)	
- Eye diseases ^c^	0.98	0.48, 2.04	0.966		(Not included)	
- Cancer	0.70	0.26, 1.85	0.472		(Not included)	
- Dementia	1.07	0.48, 2.42	0.861		(Not included)	
Pre-fracture ambulation with gait aids	2.77	1.76, 4.35	<0.001	2.33	1.32, 4.12	0.004
Hypoalbuminemia	2.17	1.39, 3.40	0.001	1.58	0.91, 2.74	0.108
Concomitant fracture	18.12	5.87, 55.98	<0.001	35.03	9.23, 132.93	<0.001
Second hip fracture	1.85	0.88, 3.88	0.105		(Not included)	
Fixation surgery ^d^	1.48	0.89, 2.49	0.134		(Not included)	
Time delayed from admission to surgery > 48 h	3.99	1.41, 11.27	0.009	2.28	0.72, 7.24	0.162
Anesthesia time (every 1 h)	1.64	1.16, 2.32	0.005	1.12	0.70, 1.77	0.641
Intra-operative blood loss (every 100 mL)	1.03	0.88, 1.22	0.695		(Not included)	
Post-operative ICU admission or ventilator use	10.58	4.62, 24.23	<0.001	6.03	2.21, 16.47	<0.001
Post-operative sedative drug use	1.86	1.18, 2.93	0.008	1.35	0.75, 2.41	0.316
Post-operative blood transfusion	1.31	0.81, 2.11	0.273		(Not included)	
Urinary catheter use on the second day post-operation	3.63	2.29, 5.74	<0.001	2.62	1.46, 4.71	0.001
Moderate to severe pain on the rehabilitation day (PS = 4–10)	0.58	0.27, 1.26	0.169		(Not included)	

Univariable and multivariable binary logistic regression presenting univariable odds ratio (uOR) and multivariable odds ratio (mOR). ^a^ Included patients with drug abuse; ^b^ COPD = chronic obstructive pulmonary disease; ^c^ eye diseases included blindness, cataracts, and glaucoma; ^d^ fixation included multiple screw fixation and dynamic hip screw fixation (DHS).

**Table 3:** Best multivariable clinical predictors for assigned item scores.

Characteristics	mOR	95%CI	*p*-Value	β	Score
Male	3.31	1.88, 5.83	<0.001	1.20	1.5
ESRD	7.31	3.11, 17.15	<0.001	1.99	2.0
Cerebrovascular disease	3.44	1.64, 7.24	0.001	1.24	1.5
Psychiatric disorders	4.58	1.64–12.76	0.004	1.52	1.5
Pre-fracture ambulation with gait aids	2.61	1.49–4.55	0.001	0.96	1.0
Concomitant fracture	40.77	11.66, −142.62	<0.001	3.71	4.0
Post-operative ICU admission or ventilator use	6.57	2.42–17.86	<0.001	1.88	2.0
Urinary catheter use on the second day post-operation	2.81	1.57–5.02	<0.001	1.03	1.0

Abbreviations: ESRD, end-stage renal disease; ICU, intensive care unit; mOR, multivariable odds ratio.

**Table 4:** Prognostic accuracy of each score risk category.

Risk Categories	Unable to Bear Self-Weight at Discharge (*n* = 99)	Able to Bear Self-Weight at Discharge (*n* = 410)	Sensitivity (%, 95% CI)	Specificity (%, 95% CI)	PPV (%, 95% CI)	LR (95% CI)	*p*-Value
	*n* (%)	*n* (%)					
Low (0–1)	14 (5.86)	225 (94.14)	92.93 (86.00, 97.10)	36.60 (31.90, 41.50)	5.86 (2.88, 8.83)	0.26 (0.16, 0.42)	<0.001
Moderate (1.5–3.5)	46 (21.20)	171 (78.80)	85.90 (77.40, 92.00)	54.90 (49.90, 59.80)	21.20 (15.76, 26.64)	1.11 (0.88, 1.42)	0.228
High (≥4)	39 (73.58)	14 (26.42)	39.40 (29.70, 49.70)	96.60 (94.30, 98.10)	73.58 (61.72, 85.45)	11.54 (6.53, 20.40)	<0.001
Median (IQR)	3 (2.5–5)	1 (0–2)					<0.001 ‡

‡ Mann–Whitney U test, CI = confidence interval, IQR = interquartile range, PPV = positive predictive value, LR = likelihood ratio.

**Table 5:** Methodological characteristics of previously reported clinical scoring systems for prediction of post-operative ambulatory status in patients with femoral neck fractures.

Scoring	Country (Sample Size)	Domain	Predictors	Endpoint
Simple scoring system [17]	Japan (323)	Femoral neck or trochanteric fracture	Anemia, dementia, abnormal lung function	Ambulation status at discharge
Six risk scores [23]	Spain (232)	Femoral neck or trochanteric fracture	RISK-VAS, Barthel, Goldman, POSSUM, Charlson, ASA	Ambulation status at 90 days
Prognostic model predicting recovery of walking independence of elderly patients after hip-fracture surgery [41]	Italy (398)	Femoral neck or trochanteric fracture	Age, gender, BMI, the number of drugs being taken, type of surgery, MMSE, pre-fracture Barthel index, IADL	The Barthel index ambulation subscore and total Barthel index at discharge
Predictive model of gait recovery at one month after hip fracture [16]	Spain (25,607)	Femoral neck or trochanteric fracture	Age, pre-fracture gait independence, cognitive impairment, ASA, fracture type, operative delay, early post-operative mobilization, weight bearing, presence of pressure ulcers, and destination at discharge	Recovery of the previous level of walking ability at 1 month
Clinical score by Tangchitphisut et al. (the present study)	Thailand (509)	Femoral neck fracture	Gender, end-stage renal disease, cerebrovascular disease, psychiatric disorders, pre-fracture ambulation with gait aids, concomitant fracture, post-operative ICU admission or ventilator use, urinary catheter use at second day post-operation	Ambulation status at discharge

Abbreviations: RISK-VAS, visual analogue scale for risk; ROC, receiver operating characteristic; POSSUM, physiological and operative severity score for the enumeration of mortality and morbidity; IADL, instrumental activities of daily living; ASA, American Society of Anesthesiologists’ Physical Status Classification; ICU, intensive care unit.

## Data Availability

All data are available upon reasonable request to the corresponding author.
